# “A chance to open my heart”: Protective factors for mental health in left-behind family members of Nepalese labor migrants and their implications for future psychosocial interventions

**DOI:** 10.1371/journal.pgph.0006459

**Published:** 2026-05-20

**Authors:** Joyce Jang, Megan Nguyen, Elizabeth Dura, Maisoun Rashed, Durga Rijal, Parbati Panday, Anup Adhikari, Nagendra P. Luitel, Pamela J. Surkan

**Affiliations:** 1 Department of International Health, Johns Hopkins Bloomberg School of Public Health‌‌, Baltimore, Maryland, United States of America; 2 Herbert Wertheim School of Public Health & Human Longevity Science, University of California San Diego, California, United States of America; 3 Prime Nepal, Chitwan, Nepal; 4 Transcultural Psychosocial Organization Nepal. Baluwatar, Kathmandu‌‌, Nepal; The University of Newcastle Australia: University of Newcastle, AUSTRALIA

## Abstract

Labor migration is prevalent in Nepal, however, the psychosocial impacts on left-behind families remain underexplored. This study aims to address this gap by examining the lived experiences, coping mechanisms, and social dynamics of left-behind family members in Chitwan District, Nepal. This qualitative study employed a modified phenomenological approach, incorporating in-depth interviews (n = 18) and two focus groups (n = 11) with left-behind family members in Nepal. Transcripts were coded and categorized using Dedoose, facilitating the identification of themes related to the experiences of left-behind family members. Findings revealed that left-behind family members engaged in activities such as household chores, religious practices, physical exercise, creative pursuits, and social media use to cope with the absence of their migrant relatives. Emotional responses included gratitude, loneliness, grief, and anxiety. A significant shift in responsibilities was noted, particularly for left-behind spouses and first-born children, who often assumed traditional paternal roles within Nepalese society. Some families experienced improved financial stability, while others faced greater stigma and social isolation. Resilience was a recurring theme, with many participants demonstrating remarkable adaptability despite the challenges that arose from family separation. Our study suggests that left-behind family members experienced important changes in their roles as well as in emotional and social relationships. Future studies could extend this work to identify factors leading to changes that promote resilience.

## Introduction

### Scientific background and rationale‌‌

Labor migration is a prevalent economically driven phenomenon in Nepal, with an increasing number of Nepalese citizens seeking employment abroad [1,2]. This trend is driven by various factors including limited local employment options, economic instability, and the allure of better wages in foreign countries [1–3]. Recent estimates state that approximately 25% of Nepal’s gross domestic product (GDP) is based on remittances from migrant workers, highlighting a substantial economic dependance on labor migration [3–6]. In 2023, labor migration from Asia far exceeded previous years (34% higher), increasing in Nepal by approximately 42% in 2022–2023 compared with the prior fiscal year [6]. While Malaysia (219,357), the United Arab Emirates (59,152), Saudi Arabia (55,791), and Qatar (40,517) were the most common destination countries for Nepalis, these figures are thought to be underestimates due to the movement of workers through atypical channels such as the India-Nepal border [6]. Households with domestic employment opportunities were found to have a lower likelihood of engaging in labor migration -- while those with smaller farm sizes, less access to irrigation and fewer assets had significantly increased likelihood of labor migration [2]. A Nepali migrant worker’s destination country tends to be associated with education and caste [7]. Nepali migrants with less schooling were more likely to work in the Malaysia and the Middle East, with the appeal of these countries declining with additional years of education [2,7]. Similarly, caste was related to country of destination, as workers from Janajati households were more likely to select Malaysia and the Middle Eastern countries compared to those from the Brahmin caste [2]. While labor migration provides financial benefits and improved living standards for many families, it also introduces social and emotional challenges, particularly for left-behind family members [8–10].

A growing body of literature has documented the adverse mental health impacts that labor migration has on left-behind family members [11–13]. Individuals with an absent migrant family member often report higher levels of stress, anxiety, and depression compared to those who did not experience family separation [11]. Moreover, children and adolescents in these families are particularly vulnerable, facing emotional difficulties due to the absence of one or both parents [12,14,15].

Despite these significant impacts, there remains a lack of focused research to identify strategies aimed at supporting left-behind family members [15]. While the economic benefits of labor migration are well-documented, the psychosocial costs are often overlooked. Addressing the mental health needs of these individuals is crucial for mitigating the adverse effects of family separation and promoting overall family well-being.

The impact of labor migration on left-behind family members is multifaceted, affecting their emotional well-being, social relationships, and daily responsibilities [16–18]. Research has predominantly focused on the experiences of youth, and literature on female caregivers within this context is scarce. While specific health impacts of labor migration on female caregivers, such as those pertaining to maternal and sexual health have been documented [19], there is a notable gap in research regarding the broader experiences of caregivers left behind by family members working abroad. Researchers from Nepal have identified a need to investigate strategies to mitigate the mental health problems affecting left-behind family members [20]. This study addresses these gaps by exploring the lived experiences of left-behind family members in the Chitwan District of Nepal, focusing on both caregivers and youth with a particular emphasis on their coping mechanisms. We sought to examine the experiences of both caregivers and youth in tandem to provide a greater understanding of shared experiences within a household.

### Study objectives

The purpose of this study is to 1) examine the lived experiences of left-behind family members in Nepal, and 2) to identify resilience factors and coping strategies in response to grief experienced due to family separation. We aim to then provide recommendations for future interventions targeting left-behind families in Chitwan, Nepal.

## Methods

### Ethical approval

Ethical approval was obtained from the Johns Hopkins Bloomberg School of Public Health (BSPH) (No.: 00021379) and the Institutional Review Board (IRB) and the Nepal Health Research Council (NHRC) (No.: 308–2022). Informed consent was given by all participating parents/guardians. For adolescent participants who were minors, the parent or guardian first provided written consent followed by the adolescent’s oral assent. For adult participants, written consent was obtained. Youth above the age of 18 were approached directly and were given full choice on their enrollment

### Study design

This qualitative study followed a modified phenomenological approach seeking to shed light on the experiences of Nepalese family members left behind from labor migration, their resilience factors and coping mechanisms. Participant recruitment and data collection was conducted in the Chitwan District of Nepal through a collaboration between the Nepali psychosocial research and service organization, Prime Nepal and the Johns Hopkins Bloomberg School of Public Health, between September 2022 and October 2023.

The research staff conducting these interviews had completed a Bachelors in Social Science, Health or Psychology and had at least 6 years of experience in mental health research, using both qualitative and quantitative methods. They followed a semi-structured interview guide.

### Research participants

Inclusion criteria for caregivers were individuals 25 years old and older who were the spouses, parents, or siblings of Nepalese migrant workers who had left Nepal to work in another country. For adolescents and young adults (henceforth referred to as ‘youth’), we included individuals between the ages of 14 and 25 years old who had an absent parent due to labor migration.

Data collection consisted of 18 in-depth interviews and 2 focus groups with a total of 11 participants (Table 1). The sample size was guided by the principal of thematic saturation. We concluded that our sample size provided sufficient depth and richness of information to adequately address the research questions.

**Table 1:** Participant demographics.

Study ID	Age	Sex	Relative Abroad	County Relative Moved To	Years Since Separation
Caregivers					
CI01	56	F	Husband	Saudia Arabia before, now Qatar	10-11
CI02	59	F	Husband	Qatar	4
CI03	39	F	Husband	Qatar	15+
CI04	36	F	Husband	Oman	15
CI05	38	F	Husband	Saudi Arabia	~17
CI06	31	F	Husband	Saudi Arabia	12
CI07	51	F	Husband	Saudi Arabia	7-8
CI08	42	F	Husband	Israel	3
CI09	65	F	Husband and Son	Husband: Japan Son: Unknown	Husband: 16–17
Focus Group Participants					
FGD1 – P1	54	F	2 Daughters	Daughter 1: U.S.A. Daughter 2: U.S.A.	Daughter 1: 4+ Daughter 2: < 1
FGD1 – P2	62	F	Son	Japan, U.S.A.	8
FGD1 – P3	52	F	Daughter	U.S.A.	6
FGD1 – P4	53	F	Son	Qatar	6
FGD1 – P5	57-58	F	Son	Denmark	1.5
FGD2 – P1	46	F	Husband	Saudi Arabia	16
FGD2 – P2	45	F	Husband	Oman	15
FGD2 – P3	67	F	Son	Saudi Arabia	16
FGD2 – P4	45	F	Husband	India	17
FGD2 – P5	65	M	2 Sons	Son 1: Qatar Son 2: Australia	Son 1: 11 Son 2: 15
FGD2 – P6	67	M	Son and Daughter	Son: U.S.A. Daughter: U.S.A.	Son and Daughter: 7
Youth					
AI01	21	F	Father	Japan, U.S.A.	8-9
AI02	19	F	Parents & Brother	Parents: Japan Brother: Australia	Parents: 8 Brother: < 1
AI03	20	F	Parents	Canada	13
AI04	15	M	Father	Saudi Arabia	13
AI05	19	F	Parents	Japan	Mother: 7–8 Father: > 8
AI06	17	F	Father	Bahrain/Romania	11
AI07	23	F	Father	Qatar/Saudi Arabia	21
AI08	23	F	Father and Sister	Father: Hong Kong Sister: Unknown	Father: 8–9
AI09	24	M	Father	United Arab Emirates	14

Data collection took place in the Chitwan District, with many participants hailing from Bharatpur Metropolitan City, the largest city within the Chitwan district that is a hub of education, health care and transportation, boasting a rising population from 579,984 in 2011 to 722,168 people in 2021 [21,22]. Labor migration has played a significant role within Chitwan. It was reported to be the 10^th^ most common origin district in Nepal for international labor migration according to the 2021 National Population and Housing Census [23]. The census reported 65,164 persons originating from the Chitwan district engaged in labor abroad with the largest proportions traveling to Middle Eastern countries (39.8%), Pacific Ocean Region countries (13.6%), or to India (11.1%) [23]. Chitwan was selected not only for its large proportion of international labor migrants, but also because of our existing collaboration with investigators associated with Prime Nepal, which was based in this district. Prime Nepal’s established relationship with the community provided a foundation for engaging respectfully and effectively with the study population.

Female Community Health Volunteers (FCHVs) identified community members who met the inclusion criteria in Chitwan. FCHVs are a vital component of many national maternal and child health programs in Nepal such as the National Immunization Program, Birth Preparedness Package, and Family Planning Program [24]. One FCHV is responsible for approximately 150 households each and they were incorporated into this study due to their close connections to the Chitwan community. Participant recruitment was conducted purposively with the assistance of the FCHVs. Once a household was identified by an FCHV, potential participants were formally approached by research personnel from Prime Nepal. In terms of sampling, FCHVs may have been more inclined to identify households with whom they maintained a pre-existing positive relationship, or those they perceived as being more open to research. This sampling method was chosen because it facilitated access and trust within the community, promoting information-rich interviews and discussion of sensitive topics related to mental health and family dynamics.

### Positionality statement

Part of our team was composed of graduate students from Johns Hopkins University. These team members came from various racial backgrounds (East Asian, Southeast Asian, Caucasian, North African) yet shared the common experience of growing up in the US and Canada and having the privilege to pursue advanced degrees. This collective background informs our approach to research, providing both strengths and potential biases. This includes a North American perspective, including assumptions about the universality of certain social and cultural norms. We are aware that our backgrounds may influence our interpretation of data and interactions with participants. Other members of our team were from Prime Nepal, a psychosocial organization located in Chitwan, Nepal. Nepali team members who conducted the interviews and participated in the interpretation of the data were predominantly female Nepalese nationals residing in Chitwan. They ensured cultural sensitivity and relevance in our research. Feedback, analysis, and diverse perspectives were provided by local staff who were from a mix of early-career and senior staff between the ages of 20–50 years old, living near the Bharatpur city area. Apart from one student pursuing a Bachelor’s degree, the rest of the team providing feedback had already obtained their Bachelor’s and were experienced in psychosocial program delivery. As a team, we critically reflected on biases inherent in the research team and took steps to minimize their impact. Reflexivity was practiced through debriefing meetings where the international and local teams discussed the interpretation of the data. These dialogues served as a critical check, challenging the international team’s assumptions and providing more context for clarity (regarding points that needed explanation sometimes because they were only understood implicitly by the local team). This collaborative process helped to ensure that cultural nuances were accurately portrayed in our results and fostered accurate interpretation of the data. Through ongoing reflexivity and collaboration between international and local partners, we aimed to consciously center the authentic voices of our Nepali participants.

### Procedures

Data collection spanned from September 12, 2022 until October 30, 2023 in partnership with Prime Nepal. Data collection was conducted by two research personnel and consisted of approximately 60-minute to 90-minute semi-structured in-depth interviews and two focus groups. One focus group was with only women and the second group consisted of both men and women. Participant demographic information was self-reported on age, gender, education level, relation to the migrant worker, and caste.

All interviews and focus groups were conducted in Nepali by trained staff from Prime Nepal. With consent from participants, all sessions were audio-recorded. The audio files were then transcribed in Nepali and subsequently translated into English by the same bilingual team members who conducted the interviews and who were deeply familiar with the project’s context and objectives.

Data analysis was conducted using the qualitative software program, Dedoose [25]. Interview transcripts were translated into English by members of the Prime Nepal research team who were fluent in Nepali and English. We employed a hybrid inductive and deductive thematic analysis approach. A preliminary codebook was developed *a priori* based on existing literature on labor migration and mental health, structured using Bronfenbrenner’s Social Ecological Model as a conceptual framework [26]. Using this structure, we initially organized codes into four domains: the migrant, the left-behind individual, interpersonal relationships, and societal factors, reflecting the micro- meso- and macrolevels identified by Bronfenbrenner. Coding for each interview was completed independently and compared across three team members (JJ, MN, ED). The data analysts met regularly to compare their coding, discuss discrepancies, and reach a consensus to modify or add codes to the codebook. This consensus-based process was repeated until the codebook was finalized and applied to all transcripts. Using this method, final codes were then analyzed to derive major themes.

While the line-by-line coding of the translated transcripts was conducted by JJ, MN and ED, Nepalese investigators played a pivotal role in the interpretive and validation stages of the analysis. Following initial coding, MR held collaborative debriefing sessions with Nepali team members and reviewed emerging themes and overall thematic structure. During these sessions, the local partners served as cultural experts and key informants. They provided critical feedback on our interpretations, confirmed or challenged the meaning of concepts, and offered nuanced explanations of the cultural context that may have been missed. This process of collaborative interpretation was essential for ensuring the final themes accurately represented the lived experiences and cultural perspectives of the participants, enhancing the credibility of the findings.

## Results

### Participants

As we incorporated the voices of youth and caretakers alike, participants recruited and included in the analysis were between 15 and 65 years of age. Participants were predominantly female (18/20 caregivers and 7/9 youth). There was a wide variation in education level, countries to which the migrants had traveled, and time since the migrant had been away. All participants however prescribed to the Hindu religion, and almost all were of the Brahmin caste, except for two participants (one who was from Terai caste and one who was from Janajati caste).

To characterize the migration context of our sample, we documented the relationship of the migrant family member to each participant. All caregivers interviewed individually were women (9/9), either with their husbands or sons working abroad. Of our youth participants, 67% had their fathers abroad (6/9), while the remaining 33% had both parents working abroad (3/9). In the focus group discussions, the most common migrant family member was a son (6/11), followed by either a daughter (3/11) or a husband (3/11). One participant had both a son and daughter abroad. Overall, within our sample, migrants were predominantly male (husbands, fathers, and sons), which is consistent with broader national trends in Nepal [23]. However, the presence of female labor migrants in our sample highlights the increasingly diverse nature of contemporary working patterns.

### Experiences of left-behind family members

#### Emotions evoked by labor migration.

The emotions evoked by labor migration and losing a family member described were far-reaching but varied greatly. Participants frequently reported feelings of gratitude, loneliness, sadness, worry, grief, and jealousy towards those who did not have migrant family members.

“When my daughter went, I felt like I needed to hang [onto] the plane. [Before,] I was crying thinking my daughter didn’t get a chance to go abroad. Now, I am crying thinking she is going. I cried so God would give my daughter a chance to go abroad. If I cry again, then maybe she will return.” - FGD01 – P1 (Female; 54 years old).

“I would like to thank him [the participant’s father] for all his hard work. Because of his hard work, we are standing here. Our father gave up everything, even his goals, to provide us with a good education and the skill to speak in front of others. I would like to thank him for everything he has done for us.” – AI09 (Male; 24 years old)

Beyond the quotes themselves, the study team noted many participants needing to take a rest between conversations, or instances when words were shared through tears.

#### Changes in personal responsibilities.

Many participants reported changes in the personal responsibilities of household members following the departure of the migrant worker. The impact appeared to be significant among the wives left behind and the eldest children of the household.

“In the absence of my father, my mother fulfilled all his responsibilities. Since my father left, my mother has fulfilled the responsibilities of both father and mother. She raised us, fulfilling every responsibility. Because of that, I have great respect for her.” – AI09 (Male; 24 years old)

Mothers often took on responsibilities that were traditionally assumed by fathers within Nepalese society. In addition to their regular responsibilities as caregivers, mothers described that they were also expected to take the children to the hospital when sick, accompany the children to school on special days, complete agricultural work, and discipline the children.

#### Changes in social relationships.

There was an apparent schism in how relationships with the community affected how labor migration had impacted the left-behind family members. Many participants reported that financial security resulting from labor migration led to moments when community members treated the family with more respect and were more willing to engage with them. On the other hand, others reported they were sometimes more isolated following the departure of the migrant worker.

“There is a positive change in our family life. Since my dad went abroad, our financial condition and family status have improved. Talking about the negative, we must face humiliation from society. We didn’t get help from relatives and neighbors in the absence of my dad. We weren’t given help when we were sick, or our animals got sick. Our relatives and neighbors used to visit us when my dad was with us, but they stopped seeing us after he left. When my friends used to humiliate me, I used to imagine that if I had my dad with us, I would share it with him.” AI08 (Female, 23 years old)

“If he [the participant’s father] were with us, then nobody could humiliate us. There is a huge difference between being together and him being away. When he is away from us, we can’t take immediate action when somebody humiliates us. In these times, I feel very low as I can’t reply to them. If my father were with us, then they wouldn’t be able to humiliate us directly.” – AI07 (Female; 23 years old)

While family members or neighbors had once visited often, participants explained how they lost this connection when one parent left. Additionally, youths noted that the country the migrant parent went to was of great significance as it may result in embarrassment or humiliation among their classmates.

“I feel they are humiliating us as he is in a gulf country. I feel if we were together, there would not be conflict and misunderstanding.” – AI07 (Female; 23 years old)

#### Difficulties in unexpected circumstances.

When discussing the experiences that left-behind family members have had without the migrant family member, notable events would often be mentioned. These events included but are not limited to times of sickness, pregnancy or labor, festival seasons, the Covid-19 pandemic, family death and mourning, and celebrations such as weddings.

“There is no one to take us to the hospital when we are sick. If we are in [the] hospital, then there is no one to take care of us. Out of the two of us [parent and child], the one who got sick, had to be taken to the hospital. No matter what happens, we must do our farming and cooking. We need to look here and there. This is a big problem for us.” - CI09 (Female; 65 years old)

In this scenario where the mother and child remained in Nepal without the father, it sometimes became difficult for the mother to accompany the child to the hospital in times of sickness due to agricultural responsibilities that pertained to their livelihood.

### Coping mechanisms

#### Engaging with household chores and other tasks.

The act of engaging oneself with other tasks as a coping mechanism was discussed many times throughout the interviews. It was mentioned both as a method to temporarily forget or overcome the loss of a family member due to labor migration and as a motivator in pursuing success academically or pushing through one’s responsibilities. Some of the tasks mentioned included: turning towards personal duties such as cooking, cleaning, or studying; centering oneself through religion and spirituality; listening to music or expressing oneself through exercise and dancing; writing poems or reading literature; and spending time on social media.

“Sometimes I go to the farm and different places to forget my pain and stress.” – CI09 (Female; 65 years old)

[In response to the question: What do you do when you are upset]

“I listen to music. I turn [on the] TV and dance.” – AI03 (Female; 20 years old)

#### Turning to religion or spirituality.

For many left-behind family members, religion and spirituality appeared to alleviate some of the stress of not wanting to burden others. Due to difficulty in sharing one’s struggles, prayer was often described as serving as a means to give this internal struggle an outlet. A focus group participant shared how she turned to religion when she struggled with her grief and loneliness of not being able to share her burdens with others:

“When I am anxious, I go to the prayer room and sit, praying to god to make everything fine. Buddha used to do the same. We didn’t need to hear bad news. We only have happiness in our life. When I think of crying, then, I sit next to god. I thank god for giving us happiness and eliminating our pain. If I share with my children, they will cry and feel tense. If I share with my husband, then he says you are making yourself unnecessarily anxious. So, I share with god. After sharing with god, I feel peace of mind and heart.” FGD01 – P1 (Female; 54 years old)

#### Maintaining a relationship with the migrant family members.

Another important coping mechanism was maintaining communication with the absent migrant family member, whether this was through calls, text messaging, or video calling. Participants shared how calls with the migrant family member were impactful in alleviating feelings of longing and sadness:

“I spoke all my heart’s feelings on the phone with him… When I miss [him], I talk on the phone to share my feelings and then the problem will be resolved.” FGD02 – P1 (Female; 46 years old)

As mentioned by some of the grandparents in our study, this has been a gift afforded through technological advancements of recent decades that weren’t as readily accessible in their youth. However, there are also specific technological challenges that the elderly generation continues to face due to poor eyesight and difficulty navigating devices. Additionally, while new avenues for communication have erased many barriers, there were other obstacles that could not be relieved such as time differences. This factor depended on where the absent family member was living, and had a crucial role in the degree to which family members could engage with one another due to scheduling conflicts.

Youth had contrasting opinions on their experiences with calls or texts from migrant parents. Some who spoke positively about their calls and texts with their parents as it opened an avenue for connection while others expressed difficulty in finding a deeper relationship. Their level of engagement through their devices appeared to be heavily dependent on their prior relationship with the migrants before labor migration.

### Influence of social support

#### Family support.

The family as a social unit was introduced not only as a financial pillar but one that also carried emotional and psychological weight. The family was often mentioned as a support system one could turn to for help with household tasks and in sharing one’s needs.

The relationship that mothers had with their children was a key source of support. Specifically, the relationship between mothers and daughters came up often. It was clear that there were many relationships where this connection between mother-daughter was often the only emotional solace that female caregivers had in the absence of their migrant spouse. This strengthened bond between mothers and daughters was a recurring theme triggered by the departure of fathers, highlighting the pivotal role daughters played in providing comfort and alleviating the emotional burden faced by their mothers.

“No matter what happens, I only share with my daughter. Nothing will happen. [If I] share with outsiders, then it will be better to remain silent.” CI01 (Female; 56 years old)

### Support from friends

Youth often highlighted the critical role of friendships in the face of family separation. Many participants described their friends as a primary source of emotional support during challenging times. This support manifested in several ways, including providing a safe space to express frustrations, offer consolation, and share worries.

“Sometimes when I feel very lonely, sometimes [when] I am anxious and my marks do not come [out] well in exams, [it] makes me sad. But my friends encourage [me] saying, ‘You should not be afraid, I am with you, and I know you well and [you will] do fine.” – AI05 (Female; 19 years old)

### Neighbors and community members

The wider social network had both positive and negative impacts on the participants. Some participants reported relief by comparing their circumstances with those of others who had been less fortunate within the community such as those who were struggling to find a stable source of income. Additionally, many found comfort by turning to friends and neighbors at social events such as festivals and weddings.

“People in my community are very helpful. We get help from the community if we ask them when we have a puja or marriage in our house. We also do the same [for them].” CI07 (Female; 51 years old)

Yet, difficulties were described concerning the wider social network, in contrast to its role as a coping mechanism. Participants shared that their hardships were reinforced when they felt they lacked social support and explained how they compared themselves to families who did not need to separate due to labor migration.

“I didn’t have anybody to share my pain and sorrows. I can’t tell it to the wall or to the people of the village.” CI02 (Female; 59 years old)

These feelings of social isolation and being unable to share one’s hardships appeared to exacerbate the difficulties left-behind family members might feel. This sense of isolation calls attention to the profound impact of labor migration on elderly parents, who often find themselves navigating the challenges of aging and daily life without the social network traditionally provided within the immediate family.

“My son says he will come this year and again he says I will go next year but he has not come. He has not come here thinking his work is good there. But we want our son to return as we are old. If our son and daughter-in-law stay with us, it will be easy for us. We [participant and her husband] can’t go and work now. So, if they are with us, then it will be easy for them as they will prepare the food. We are worried, thinking about if we died without seeing them. I have seen such situations.” CI09 (Female; 65 years old)

#### Inner strength.

Inner strength, which grants people the ability to cope and thrive when faced with hardship, was expressed by multiple participants who shared their struggles.

“What could I do? I just consoled myself, thinking it was [a] part of life and when I felt [it was] most difficult, I used to cry and that would calm me. Then, I would get up and face my responsibilities.” - CI06 (Female; 31 years old)

#### Sharing through times of struggle.

There was a general consensus of gratitude for being able to share the experiences that resulted from labor migration. Many participants, whether they were involved in in-depth interviews, or focus groups, or were caregivers or youth, had expressed relief in being able to share their experiences and feelings.

“Thank you too, as you gave me a chance to open my heart.” - CI01 (Female; 56 years old)

However, there were recommendations that came from our focus group discussions where participants suggested the psychosocial organization that facilitated these conversations to continue helping those who are in need in the community and to reach out to those who are living abroad.

“Everything that has been discussed, what you and your organization are doing is commendable. You ask for help from people who can give it and give help to needy people who are unable to bear treatment expenses, unable to return home, and have difficulties. I want your organization to help manage people in need living abroad as all people aren’t lucky living abroad.” – FGD02 P5 (Male; 65 years old)

In addition to the positive responses to the focus groups, one participant mentioned appreciation at being able to discuss these topics in a mixed group of males and females.

“I would like to thank you and Prime Nepal for this opportunity. I would like to thank all participants. In the past, you have done this kind of program before. I feel like it’s a good idea to mix males and females. Because of that, we had a chance to listen to each other. We got the opportunity to learn from each other’s point of view and how we all feel.” – FGD02 P6 (Male; 67 years old)

#### Advice for other families.

Our data suggested a sentiment that youth who now consider leaving Nepal for migrant work should first consider opportunities in Nepal. Participants suggested making sure to carefully consider the purpose of going and if there is work available at home, to stay home. Worries regarding the future development of the country as many young Nepalese men and women leave for work abroad have taken hold. Even for those who are currently abroad, many participants suggested considering coming back if they are able to, that it would be better to stay with their family and earn less than to leave and earn more.

“I want to say 'don’t go abroad if you can' to the youth who are planning to go. Just work in Nepal and utilize your skills here, as a country abroad is not the same as our native one. If you are abroad, the family is separated. It is not possible to return to your country when you want and when there is an emergency. Don’t think about going abroad. Stay in your own country and do what you can. But if the youth who are responsible for building the country are going abroad, then how will our country become better? Don’t go abroad and don’t make your parents cry. Without you, your parents will suffer a lot, and you will suffer after going abroad.” - CI09 (Female; 65 years old)

However, many community members recognized that staying isn’t a luxury that all can afford, and there are circumstances where family members must leave for work to provide for their families. For the families where migrant work is necessary, participants encouraged others to focus on contact between family members and shared their struggles with one another.

“I want others to understand that their family members have gone abroad for them. They have gone out of compulsion; it is not their wish to go abroad. We should not be dependent on others for life.” - AI08 (Female; 23 years old)

Lastly, many adult participants advised youth to understand that parents often do not leave because they want to, but rather it is to provide their children a better future so that they may live happy and comfortable lives.

## Discussion

### Main findings

The emotional impact of labor migration was profound. The lived experiences within the left-behind family members displayed a wide variety of emotions, shifts in personal responsibilities, and changes to social relationships. Findings suggest that the hardships that came with labor migration were often mitigated through five main coping strategies: 1) engaging with other tasks; 2) maintaining a relationship with the migrant; 3) social support; 4) inner strength and resilience; and 5) sharing in times of struggle.

We found that commonly reported emotions arising from labor migration included gratitude, loneliness, grief, and anxiety. Several other studies within the Southeast Asian context, have similarly highlighted the emotional and social impacts of labor migration on left-behind families, including the psychological strain and the shift in family dynamics. Some emotional responses to family separation and the absence of parents among children have included feelings of loneliness, abandonment, anger, confusion and anxiety [27]. For example, Fauk and others found separation from parents due to labor migration was detrimental to left-behind children’s social and mental well-being in Indonesia, as they faced negative perceptions from peers and experienced changing household dynamics [17]. Similarly, in Thailand, Penboon and Jampaklay reported that the strain of labor migration was associated with mental health problems and subsequent behavioral concerns in children [28]. Kumar highlighted the effects of labor migration on older parents in Indonesia, showing heightened risks for depression and general distress [29]. Broadening the scope to Asia as a whole, one study in China that looked at the developmental consequences of parent-child separation found that while there were no overall significant associations between parental migration and social-emotional development, there was an association between maternal migration and social-emotional problems in children [30]. Lastly, Mahat and colleagues’ study in Nepal found a high prevalence of mental health conditions such as anxiety, depression, and suicidal ideation among both migrant workers and left-behind family members [20].

One of our main findings was that participants relied on daily activities and responsibilities as coping mechanisms. Kim and others studied the idea of self-distraction, social connection, and religion as important coping strategies during the Covid-19 outbreak [31]. They found that the use of these strategies maintained participants’ well-being in conjunction with preventive measures and behavioral strategies [31]. Within the Nepalese labor migration environment, Aryal and others found that left behind spouses who communicated with their migrant husbands at minimum once a day had more resilience, compared to those who did not [32]. The positive influence that communication with the migrant family member has on left-behind family members was also seen in our study as it provided solace and support.

While our results were organized thematically for clarity, Bronfenbrenner’s Social Ecological Model, which guided our initial inquiry, provides a powerful lens for interpreting the our findings [26]. The model underscores that interventions cannot solely target the individual. To be effective, support must be multi-level, aiming to strengthen family communication, foster community cohesion, and work within prevailing cultural contexts.

The complexities involved in such experiences of family separation emphasize the multi-faceted social dynamics faced by these families. Our main finding was that these coping mechanisms (e.g., engaging with other tasks and turning towards social support) were notable protective factors against the detrimental mental health effects of family separation through labor migration. Being able to turn to others in times of trouble, share one’s hardships, engage in other tasks and recognize the sacrifices made for the betterment of one’s family’s future were pivotal to moving forward.

### Recommendations and implications for the future

Many participants recommended that other families separated due to migration should maintain regular contact with migrant family members abroad. This could be promoted through facilitated discussions (e.g., making specific plans regarding contact methods, who to turn to in an emergency, discussing who will assume which responsibilities, and how often visits will be planned). Realistic expectations could be set that purposively include youth in the conversation, so that they may better understand their parents.

Additionally, we found that sharing one’s experiences could grant a sense of catharsis. This was emphasized through the gratitude that participants expressed at having the opportunity to hear the stories of others in the community with similar experiences through participation in a focus group. There was even desire for future discussion group events to be held, enabling the community to better understand and listen to one another. We suggest future interventions to incorporate such mixed-gender discourse and group counseling at the community level. Support may be particularly important for left-behind parents of children who have left to become migrant workers who often voiced struggles with loneliness in their advanced age. Left-behind parents were also identified as a vulnerable subgroup struggling with loneliness. Community-based interventions could promote the organization of social gatherings specifically for this group to foster connection and reduce social isolation.

Our findings suggest several initial strategies for the development and implementation of psychosocial interventions. Based on the findings, we propose a multi-pronged approach: 1) develop peer support groups; 2) implement pre-departure family planning workshops; and 3) create targeted outreach for the elderly. By employing these strategies together, a safety net could be created for left behind family members to buffer against the negative emotions and stressful situations arising from family separation. These interventions would support left-behind family members, especially vulnerable populations such as children, elderly parents, and caregivers who are parenting alone. Future research should pilot and formally evaluate the effectiveness of such programs, refining their core components based on empirical evidence. Given external constraints, the implementation of psychosocial support programs must be flexible and contextually sensitive to meet the varied needs of families affected by labor migration.

### Strengths and limitations

One strength of this study was that we interviewed both parents and youth to triangulate different perspectives. Having Nepali team members who understood the cultural-societal context, ensured that our study was culturally sensitive and the questions we asked aligned with the community’s needs and perspectives. The study’s focus on the Chitwan district in Nepal is another strength, as it is one of the districts that is most affected by labor migration [2]. By concentrating on a specific district, the research was able to provide a more detailed and nuanced understanding of the experiences of left-behind family members in this region. The Chitwan district, with its particular socio-economic and cultural characteristics, offers a strong representation of the local community affected by labor migration. While this specificity allows for in-depth analysis and meaningful insights that are highly relevant to the local context, findings may also have limited transferability to migrants from other parts of Nepal. Likewise, because participants were predominantly Brahmin and practiced the Hindu faith, the findings may not be fully applicable to Nepali migrant families from other castes or religious traditions. However, about 81% of the population in Chitwan is Hindu, making them highly representative of the population in the district [22]. While almost half of our adolescent participants had migrant fathers, there were only three participants who experienced the absence of both parents. Given recent numbers indicating a rise in female migrant workers from Nepal [33], future research should investigate the absence of both parents and the sole absence of a migrant mother. Finally, our data was collected at a single time point. Given long-term impacts of labor migration on families can evolve over time, future studies could include a longitudinal approach that could provide deeper insights.

### Summary

In conclusion, protective factors of mental health in left-behind family members of Nepalese labor migrants included various coping mechanisms, such as religious practices, creative pursuits, and social media use, in response to shifts in family roles and financial stability. This study emphasizes the need for identifying targeted psychosocial interventions that can incorporate effective coping mechanisms (e.g., facilitated family discussions, community safe spaces to support these families).

## Supporting information

S1 Checklist Checklist‌‌. (DOCX)

## References

[1] Rai N, Khadka N, Rai M, Shrestha P, Lekhak M, Shrestha M, et al. Rise of Foreign Employment and Challenges Faced by Nepali Youth in the Domestic Job Market. International Journal of Applied and Advanced Multidisciplinary Research. 2024;2(7):497–508. doi: 10.59890/ijaamr.v2i7.2140

[2] Thapa Magar DB, Pandit R, Fay Rola-Rubzen M. Drivers of overseas labour migration, migration intensity, and destination choice among farming households in Nepal. J Ethn Migr Stud. 2024;:1–25. doi: 10.1080/1369183x.2024.2373311

[3] Bastola LP. International Migration: Causes and Consequences in Nepal. Spectrum of Humanities & Social Sciences. 2025;1(1).

[4] Personal remittances, received (% of GDP) - Nepal. World Bank Open Data. https://data.worldbank.org/indicator/BX.TRF.PWKR.DT.GD.ZS?locations=NP

[5] Khatiwada PP, Basyal K. Labour Migration in Nepal: Policy, Institutions, and Governance. Molung Educ Frontier. 2022;12(01):144–72. doi: 10.3126/mef.v12i01.45922

[6] Asian Development Bank Institute, International Labour Organization, Organization for Economic Co-operation and Development. Labor migration in Asia: trends, skills certification, and seasonal work. 2024.

[7] Regmi M, Paudel KP, Bhattarai K. Migration decisions and destination choices. Journal of the Asia Pacific Economy. 2019;25(2):197–226. doi: 10.1080/13547860.2019.1643195

[8] Sharma S. Parental Migration and Psycho-Social Challenges of Left-Behind Girls in Nepal. Prithvi J Res Innov. 2023;5:37–52. doi: 10.3126/pjri.v5i1.60690

[9] Zhao C, Wang F, Zhou X, Jiang M, Hesketh T. Impact of parental migration on psychosocial well-being of children left behind: a qualitative study in rural China. Int J Equity Health. 2018;17(1):80. doi: 10.1186/s12939-018-0795-z 29903019 PMC6003177

[10] Nguyen M, Kim Y, Choi Y, Jang J, Shakya M, Adhikari A, et al. Guardians Looking From Outside: Gendered Experiences of Labor Migration and Psychosocial Health Among Nepalese Migrant Fathers and Left-Behind Mothers. Qual Health Res. 2025;35(6):665–79. doi: 10.1177/10497323241265291 39293817

[11] Kharel M, Akira S, Kiriya J, Ong KIC, Jimba M. Parental migration and psychological well-being of left-behind adolescents in Western Nepal. PLoS One. 2021;16(1):e0245873. doi: 10.1371/journal.pone.0245873 33507904 PMC7842897

[12] Fellmeth G, Rose-Clarke K, Zhao C, Busert LK, Zheng Y, Massazza A, et al. Health impacts of parental migration on left-behind children and adolescents: a systematic review and meta-analysis. Lancet. 2018;392(10164):2567–82. doi: 10.1016/S0140-6736(18)32558-3 30528471 PMC6294734

[13] Zheng J, Yan L. The impact of left-behind experience on the mental health and marital satisfaction. Front Psychol. 2025;16:1603281. doi: 10.3389/fpsyg.2025.1603281 40497098 PMC12150875

[14] Fu W, Xue R, Chai H, Sun W, Jiang F. What Matters on Rural Left-Behind Children’s Problem Behavior: Family Socioeconomic Status or Perceived Discrimination. Int J Environ Res Public Health. 2023;20(2):1334. doi: 10.3390/ijerph20021334 36674091 PMC9859111

[15] Račaitė J. Emotional and behavioural problems of left behind children in Lithuania: A comparative analysis of youth self-reports and parent/caregiver reports using ASEBA. Child and Adolescent Psychiatry and Mental Health. 2024;18(33). doi: 10.1186/s13034-024-00726-y PMC10949819 38500119

[16] Démurger S. Migration and families left behind. 2015. 10.15185/izawol.144

[17] Fauk NK, Seran AL, Aylward P, Mwanri L, Ward PR. Parental Migration and the Social and Mental Well-Being Challenges among Indonesian Left-Behind Children: A Qualitative Study. Int J Environ Res Public Health. 2024;21(6):793. doi: 10.3390/ijerph21060793 38929039 PMC11203627

[18] Hoang LA, Lam T, Yeoh BSA, Graham E. Transnational migration, changing care arrangements and left-behind children’s responses in South-east Asia. Child Geogr. 2015;13(3):263–77. doi: 10.1080/14733285.2015.972653 27134570 PMC4837485

[19] Shattuck D, Wasti SP, Limbu N, Chipanta NS, Riley C. Men on the move and the wives left behind: the impact of migration on family planning in Nepal. Sex Reprod Health Matters. 2019;27(1):1647398. doi: 10.1080/26410397.2019.1647398 31533579 PMC7887959

[20] Mahat P. Mental health problems in Nepalese migrant workers and their families. Journal of BP Koirala Institute of Health Sciences. 2021;4(1):64–7. doi: 10.3126/jbpkihs.v4i1.36081

[21] About: Chitwan District. https://dbpedia.org/page/Chitwan_District

[22] National Population and Housing Census. National Population and Housing Census. 2024. https://censusnepal.cbs.gov.np/results

[23] National Statistics Office. International migration in Nepal – thematic report VI. Kathmandu: National Statistics Office. 2025.

[24] USAID JSIR& TI. Nepal Family Health Program Technical Brief #1 Female Community Health Volunteers. https://publications.jsi.com/JSIInternet/Inc/Common/_download_pub.cfm?id=12140&lid=3

[25] SocioCultural Research Consultants L. Dedoose Version 9.0.17. Cloud application for managing, analyzing, and presenting qualitative and mixed method research data. 2021. https://www.dedoose.com

[26] Bronfenbrenner U. Ecological models of human development. International Encyclopedia of Education. Oxford, England: Elsevier. 1994.

[27] Parreñas RS. Children of Global Migration: Transnational Families and Gendered Woes. Stanford University Press. 2005.

[28] Penboon B, Jampaklay A. The impact of transnational labor migration on mental health of left-behind family: Care drain and care chain situation in Thailand. Mahidol University: Institute for Population and Social Research. 2017.

[29] Kumar S. The hidden costs of mobility: changing patterns of labor migration and health implications for left behind families in Indonesia. Cornell University. 2021.

[30] Shi H, Wang Y, Li M, Tan C, Zhao C, Huang X, et al. Impact of parent-child separation on children’s social-emotional development: a cross-sectional study of left-behind children in poor rural areas of China. BMC Public Health. 2021;21(1):823. doi: 10.1186/s12889-021-10831-8 33926397 PMC8082618

[31] Kim JH, Shim Y, Choi I, Choi E. The role of coping strategies in maintaining well-being during the COVID-19 outbreak in South Korea. Soc Psychol Personal Sci. 2022;13(1):320–32. doi: 10.1177/1948550621990595

[32] Aryal N, Regmi PR, van Teijlingen E, Trenoweth S, Adhikary P, Simkhada P. The Impact of Spousal Migration on the Mental Health of Nepali Women: A Cross-Sectional Study. Int J Environ Res Public Health. 2020;17(4):1292. doi: 10.3390/ijerph17041292 32079358 PMC7068335

[33] Shrestha N. Stigma and empowerment: A qualitative study on Nepalese women and labour migration. 2020. https://www.researchgate.net/publication/345227861_Stigma_and_empowerment_A_qualitative_study_on_Nepalese_women_and_labour_migration

